# Family Medicine: A profession for the world’s upper and middle class?

**DOI:** 10.4102/phcfm.v2i1.247

**Published:** 2010-11-29

**Authors:** Raymond Downing

**Affiliations:** 1 Department of Family Medicine, Moi University

## Abstract

Family medicine is a medical speciality, or at least an approach to medical care, that was developed and thrives in high-income countries. Some of the key principles of family medicine were developed in response to the disease pattern prevalent in those high-income countries – that is, the predominance of chronic, non-communicable diseases. Yet, the burden of disease in low-income countries, such as in much of sub-Saharan Africa, involves substantially more communicable disease and trauma than that in high-income countries. Consequently, the design of family medicine as developed in high-income countries may not be applicable in sub-Saharan Africa.

## THE DEVELOPMENT OF FAMILY MEDICINE

Family medicine is a medical speciality or at least an approach to medical care, that was developed and thrives in high-income developed countries. Consider the table of the human development index (HDI) of 178 countries^1^ compared with the presence of family medicine as a medical speciality (represented by an official link to WONCA^2^) in those countries (see Table 1). The HDI is a comparative measure of life expectancy, educational level and income. The table divides the countries into quartiles, depending on the index.

There is a similar pattern when considering countries according to the percentage of the population over the age of 65, higher percentages often occurring in more developed countries (see Table 2).^3^

**FIGURE 1:**
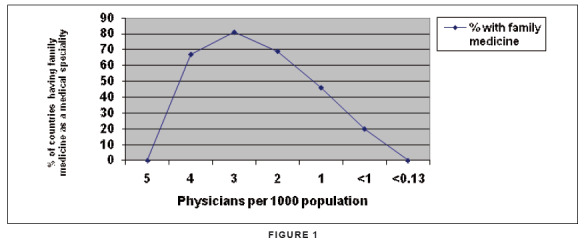


Interestingly, the few countries (about 10) with very high physician-to-population ratios (> 4 per 1000) are less likely to have family medicine compared with those having slightly less high ratios (2−3 per 1000) (see Figure 1). However, the probability of having family medicine drops rapidly when the ratio falls below 1 physician per 1000 population and none of the 32 countries with ratios less than 0.13 per 1000 (i.e. 1 doctor for 7692 patients) have family medicine.^4^

**TABLE 1:**
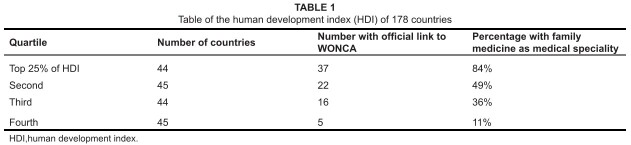
Table of the human development index (HDI) of 178 countries

**TABLE 2:**
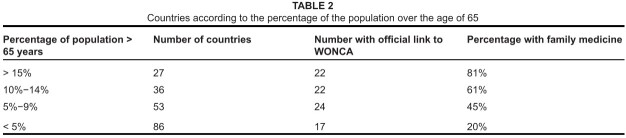
Countries according to the percentage of the population over the age of 65

## BURDEN OF DISEASE IN HIGH INCOME COUNTRIES

Some of the key principles of family medicine were developed in response to the disease pattern prevalent in those high-income countries – that is, the predominance of chronic, non-communicable diseases. In high-income countries, the disease burden is heavily due to chronic, non-communicable diseases (87%), whereas the comparable burden of these diseases in low- and middle-income countries is 54%.^5^ Likewise, chronic, non-communicable diseases increase with age, so that countries with higher proportions of people over the age of 65 will have heavier chronic disease burdens. This chronic disease burden in high-income countries is a phenomenon that developed during the first half of the 20th century.

However, because the needs of those with ongoing diseases are fundamentally different from those who have acute infectious or surgical conditions, the approach to chronic disease care must differ from the acute care model. Health care systems that do not adjust find themselves in disarray.^6^ It was in this context that some of the pivotal principles of family medicine developed, especially *continuity*, *comprehensiveness*, *coordinated* and *collaborative* care and a *bio-psychosocial* approach.

Continuity is vital when the patient must keep returning for ongoing care; comprehensiveness is a logical approach to patients with chronic conditions who often have co-morbidities. As patient problems become more complex (and the medical system develops more complex treatments), coordination and collaboration become necessary. At the same time, health education and health promotion become more important when patients must be active participants in their ongoing care. In addition, chronically ill people are more subject to psychological stresses such as depression and being able to navigate the border between psyche and soma becomes a hallmark of family medicine.

Clearly caring for chronic disease is not the *only* task of family medicine. In fact, the fundamental task is primary care: first-contact, doctor-level care. Therefore, there are many principles not related specifically to chronic disease, such as patient-centred and community-orientated care, health promotion and disease prevention and seeing all disease in its larger family and environmental context. However, a pivotal factor – perhaps *the* pivotal underlying reason – for the development of family medicine was the medical system coming to grips with the problems of chronic disease.

**The burden of disease in low-income countries:** involves less chronic disease and substantially more communicable disease and trauma, compared with that in high-income countries. These conditions, when grouped together with maternal, perinatal and nutritional deficiencies, cause about 46% of the disease burden in low- and middle-income countries – compared with only 13% in high-income countries.^5^

However, looking specifically at sub-Saharan Africa, this predominance of ‘diseases of poverty’ means the chronic disease burden is substantially lower than in the rest of the world, most likely only 15%−20%. When we consider AIDS as a chronic disease (which it is when ARVs are available) and group it with other chronic but non-communicable diseases, the chronic disease burden in sub-Saharan Africa is over 50%.^7^ Yet, because not all AIDS patients needing ARVs in sub-Saharan Africa are actually getting them (probably only about 30%^8^), the classification of AIDS as a chronic disease is not always accurate. Table 3, drawn from the previously mentioned sources, summarises this:

Projections do suggest that even in low-income countries, the proportion of the disease burden from non-communicable diseases will continue to rise with the epidemiologic transition^9^ – and that this rise may be more advanced than is generally realised. For example, the commonly repeated estimate of the prevalence of hypertension in Africa is 20 million. Yet, a recent projection based on extrapolations from current studies suggests the number is closer to 80 million (personal communication from Dr. Marc Twagirumukiza).

Nevertheless, the proportion of non-communicable disease is nowhere near the 87% of the high-income countries and will not be near it for at least another generation – or most likely, more. In addition, childhood morbidity and mortality contribute more heavily to the disease burden in poor countries, compared with rich countries. Thus, low-income countries must continue to respond to the double burden of communicable and non-communicable diseases. But the approach to chronic, non-communicable diseases, as we have seen with the development of family medicine, requires a different approach than episodic care. Thus, low-income countries must maintain two *systems* of health care – one for the Group I diseases they have always confronted and the other for the Group II diseases that are emerging there. This has implication for the nature of first-contact doctor care, or family medicine.

**The design of Family Medicine:** as developed in high-income countries may not be applicable in sub-Saharan Africa. Because family medicine is well developed in high-income countries and has established itself as the flagship of doctor-level primary care, it is now being promoted actively worldwide. Indeed, primary care is a critical part of any health system – but it must be designed with respect to the predominant disease burden in that place.

Whilst this is obvious, there is an unstated assumption that the principles of family medicine, as developed in high-income countries, are universal and can be adapted throughout the world. Certainly the principles have been tested in places where chronic disease is common and have proven themselves. But are they in fact universal, applicable to all disease patterns?

Consider, as an example, *continuity*. Anyone who has provided chronic disease care knows how difficult and time-consuming it is to first get to know the medical story of a complex chronic disease patient – yet, we would all affirm the practical and therapeutic value of *knowing* that patient and developing a continuous relationship. But what does continuity mean for acute minor traumatic wound care? Or for intensive hospital care? To preserve the principle, we try to stretch continuity to fit these categories: continuity might mean that the patient is on my panel of patients and I should know (and follow up with) the factors leading to his minor trauma. Or in a busy hospital, I cannot work day and night, so we speak of continuity of information on the same patient. Whilst these are valid applications of this principle, they highlight the difficulty of preserving a principle that does not quite ‘fit’.

Likewise, with sensitivity to psychological issues in our patients, where psychological issues are common and untreated, we must know how to recognise and manage them. But in an environment where people often deal with psychological issues through traditional means and seek our assistance mostly as biomedical technicians, our need for managing psychological problems diminishes. There is still a role for a well-developed, bio-psychosocial approach; perhaps especially in chaotic urban areas where people are separated from their roots – but in rural underserved areas, that role may be smaller.

To put all of this another way: approaching acute disease involves a different mindset than approaching chronic disease. To treat acute disease requires the clinician to take steps to ‘fix the problem’; the goal is cure, elimination of the disease. However, the clinician treating chronic disease thinks differently and has different goals: the approach is to manipulate variables in the course of the disease to reduce its severity and impact. The clinician is aiming not to cure the disease, but to *manage* it. Many of the family medicine principles developed in high-income countries reflect this approach, not only for chronic disease care, but also for community health and preventive medicine. Here, the goal is to manipulate variables in the population to reduce the impact of disease there; this is management not of a chronic disease, but of a community.

We must be clear: there is nothing inappropriate in Africa about continuity or bio-psychosocial care or any of the other principles developed in the context of a heavy chronic disease burden. But should these be the pivotal guiding principles for a place where half of the disease burden is *not* chronic disease? Clearly community orientation and context-specific care appear to be quite appropriate in low-income countries. But are we careful to always make it clear *which* principles of family medicine we are talking about in Africa?

One reason to ask this question is that there may be a sinister side-effect of uncritically adopting for Africa the principles of family medicine developed in places where the main disease burden is chronic disease. This type of family medicine will train doctors to provide excellent care for those with chronic diseases – truly a growing need. But will that training develop doctors to care equally for the large burden of communicable, traumatic, maternal and perinatal conditions and nutritional deficiencies? Or will it encourage them to work in areas where they can practise the principles they have learned, places where patients have (*and can afford to have*) chronic diseases? The valued, ongoing doctor-patient relationship is important in chronic disease care, but it is expensive. Can the chronic disease model succeed in poor communities? How will it be funded? In other words, if family medicine in sub-Saharan Africa is developed according to principles developed in high-income countries, could this result in preferentially better care for those with non-communicable diseases, possibly representing the richer segments of the population – and therefore paradoxically increase inequity?

## CONCLUSION

Family medicine developed in high-income countries in the middle of the 20th century as those countries were addressing their relatively new problem of chronic disease care. These (family medicine and chronic disease care) were parallel, but not unrelated, developments, so it was inevitable that the principles of family medicine would be drawn at least partly from this chronic disease experience. Even the chronic and recurrent depression, anxiety and psycho-physiological conditions that seem to accompany ‘development’ could be addressed by these chronic disease principles. In other words, family medicine derived its principles by addressing the predominant disease burden, which in rich countries was chronic disease. Should this approach – identifying and addressing the predominant disease burden – be any less true as family medicine develops in sub-Saharan Africa?
